# Intraoperative hypothermia and its clinical outcomes in patients undergoing general anesthesia: National study in China

**DOI:** 10.1371/journal.pone.0177221

**Published:** 2017-06-08

**Authors:** Jie Yi, Yongjing Lei, Shiyuan Xu, Yongyu Si, Shiyang Li, Zhongyuan Xia, Yisa Shi, Xiaoping Gu, Jianshe Yu, Guohai Xu, Erwei Gu, Yonghao Yu, Yanqing Chen, Hequn Jia, Yinglin Wang, Xiuli Wang, Xiaoqing Chai, Xiaoju Jin, Junping Chen, Meiying Xu, Junyu Xiong, Guonian Wang, Kaizhi Lu, Wenli Yu, Weifu Lei, Zaisheng Qin, Jingguo Xiang, Longyun Li, Ziyong Xiang, Shuang Pan, Lujing Zhan, Kai Qiu, Min Yao, Yuguang Huang

**Affiliations:** 1 Department of Anesthesiology, Chinese Academy of Medical Sciences & Peking Union Medical College Hospital, Beijing, China; 2 Department of Anesthesiology, the Second People's Hospital of Wuhu, Wuhu, Anhui, China; 3 Department of Anesthesiology, Zhujiang Hospital of Southern Medical University, Guangzhou, Guangdong, China; 4 Department of Anesthesiology, the Second Affiliated Hospital of Kunming Medical University, Kunming, Yunnan, China; 5 Department of Anesthesiology, Quanzhou Women's and Children's Hospital, Quanzhou, Fujian, China; 6 Department of Anesthesiology, Renmin Hospital of Wuhan University, Wuhan, Hubei, China; 7 Department of Anesthesiology, Lanzhou University Second Hospital, Lanzhou, Ganshu, China; 8 Department of Anesthesiology, Nanjing Drum Tower Hospital, Nanjing University Medical School, Nanjing, Jiangsu, China; 9 Department of Anesthesiology, the Affiliated Hospital of Inner Mongolia Medical University, Inner Mongolia, China; 10 Department of Anesthesiology, the Second Affiliated Hospital of Nanchang University, Nanchang, Jiangxi, China; 11 Department of Anesthesiology, the First Affiliated Hospital of Anhui Medical University, Hefei, Anhui, China; 12 Department of Anesthesiology, Tianjin Medical University General Hospital, Tianjin, China; 13 Department of Anesthesiology, Fujian Provincial Hospital, Fuzhou, Fujian, China; 14 Department of Anesthesiology, the Fourth Hospital of Hebei Medical University, Shijiazhuang, Hebei, China; 15 Department of Anesthesiology, Haikou People’s Hospital, Haikou, Hainan, China; 16 Department of Anesthesiology, the Third Hospital of Hebei Medical University, Shijiazhuang, Hebei, China; 17 Departement of Anesthesiology, Anhui Provincial Hospital, Hefei, Anhui, China; 18 Department of Anesthesiology, the First Affiliated Hospital of Wannan Medical College, Wuhu, Anhui, China; 19 Department of Anesthesiology, Ningbo No.2 Hospital, Ningbo, Zhejing, China; 20 Department of Anesthesiology, Shanghai Chest Hospital of Shanghai Jiaotong University, Shanghai, China; 21 Department of Anesthesiology, the Second Hospital of Dalian Medical University, Dalian, Liaoning, China; 22 Department of Anesthesiology, Harbin Medical University Cancer Hospital, Harbin, Heilongjiang, China; 23 Department of Anesthesiology, the First Affiliated Hospital of Chongqing Third Military Medical University, Chongqing, China; 24 Department of Anesthesiology, Tianjin First Central Hospital, Tianjin, China; 25 Department of Anesthesiology, Qilu Hospital of Shandong University, Jinan, Shandong, China; 26 Department of Anesthesiology, Nanfang Hospital, Guangzhou, Guangdong, China; 27 Department of Anesthesiology, the People's Hospital of Sanya, Sanya, Hainan, China; 28 Department of Anesthesiology, Sino-Japanese Union Hospital of Jilin University, Changchun, Jilin, China; 29 3M R&D Center, Shanghai, China; University of Pennsylvania, UNITED STATES

## Abstract

**Background/Objective:**

Inadvertent intraoperative hypothermia (core temperature <36°C) is a frequently preventable complication with several adverse consequences. Our study aimed to determine the overall incidence of inadvertent intraoperative hypothermia and its risk factors associated with clinical outcomes in this national survey in China.

**Methods:**

We conducted a national cross-sectional study with 30 days postoperative follow-up from November 2014 through August 2015. A total of 3132 eligible patients underwent general anesthesia were randomly selected from 28 hospitals in the nationwide of China.

**Results:**

The overall incidence of intraoperative hypothermia was as high as 44.3%, in which cumulative incidence rates of hypothermia being 17.8%, 36.2%, 42.5% and 44.1% within 1 h, 2 h, 3 h and 4 h respectively following induction of anesthesia. All patients were warmed passively by covering of surgical draping, sheets or cotton blankets, whereas only 14.2% of patients received active warming with space heaters or electric heater or electronic blankets. Compared to normothermic patients, patients with hypothermia is associated with more postoperative ICU admit, longer PACU and more postoperative hospital days, but no difference in surgical site infection (SSI) rates or 30-day mortality. Several factors were shown to be associated with decreased risk of hypothermia. They are active warming (OR = 0.46, 95% CI 0.26–0.81), BMI ≥ 25 (OR = 0.54, 95% CI 0.45–0.65), higher baseline core temperature (OR = 0.04, 95% CI 0.03–0.06), and higher ambient temperature (OR = 0.83, 95% CI 0.78–0.88). Risk factors associated with an increased risk of hypothermia included major-plus surgery (OR = 1.49, 95% CI 1.23–1.79), and long anesthesia (>2 h) (OR = 2.60, 95% CI 2.09–3.24).

**Conclusions:**

The incidence of intraoperative hypothermia in China is high, and the rate of active warming of patients during operation is low. Hypothermia is associated with more postoperative shivering, increased ICU admissions, and longer postoperative hospital days.

## Introduction

Intraoperative hypothermia, defined as core temperature <36.0C^o^ during surgery, is a common complication among surgical patients[1]. Hypothermia is associated with numerous adverse consequences[2–4], including postoperative cardiovascular events[5,6], perioperative hemorrhage[7,8], disturbed drug metabolism[9,10], and postoperative infection[11–15].Hypothermia may also lead to prolonged retention time in PACU or ICU [16] and decrease in thermal comfort, patient satisfaction and increasing cost [17,18]. The incidence of hypothermia was reported between 4% and 72% [19] and up to 90% by some studies [3,20]. Although many professional societies, such as Association of periOperative Registered Nurses (AORN), www.aorn.org, and National Institute for Health and Care Excellence (NICE), www.nice.nhs.uk, have made recommendations [21]for preventing perioperative hypothermia and improving its management during the perioperative period, maintaining perioperative normothermia in China is not required by the current standard of care. Our previous study [22]showed that incidence of hypothermia was as high as 39.9% in the City of Beijing, and as low as 10% of patients received active warming (space heater, electronic heating blanket, etc.) in their operations. All patients were passively warmed with cotton blankets, sheets, or surgical draping, but these measures have been reported to be ineffective in maintaining intraoperative core temperature. As City of Beijing has one of the best healthcare providers, facilities and resources, these data clearly reveal some shortcomings in our clinical practices.

Our study aimed to determine the incidence of inadvertent intraoperative hypothermia and its associated clinical outcomes in China.

## Patients and methods

### Selection of study sites

This is a national cross-sectional study with 30 days postoperative follow-up from November 2014 through August 2015. As an observational study, our study protocol was fully approved first by the Ethics Committee and Institutional Review Board (IRB) by Peking Union Medical College Hospital (PUMCH), the leading site of the entire study. Then it was accepted by all other 27 participating sites nationwide.

This study had been also registered at www.clinicaltrials.gov (National Clinical Trial (NCT) number: NCT02211703). All participants signed informed consent forms (ICF) before enrolled in the study.

### Subjects, inclusion and exclusion criteria

The study included subjects from 28 major teaching hospitals nationwide in China who underwent elective operations with an estimated duration of general anesthesia of more than 30 minutes. Participants were excluded if they had: 1) high central fever caused by cerebrovascular disease, cerebral trauma, cerebral operations, epilepsy, or acute hydrocephalus; 2) thermoregulation abnormalities (e.g., malignant hyperthermia, neuroleptic malignant syndrome); 3) infectious fever with core temperature one week before operation higher than 38.5°C; or 4) history of hypothyroidism or hyperthyroidism. Subjects also were excluded if they were younger than 18 years of age (because of parents’ concerns for the risk of tympanic membrane perforation during temperature monitoring).

### Clustered randomized sampling

According to our previous results from Beijing, the incidence rate of hypothermia was 39.9% among patients under general anesthesia lasting over 60 minutes. We use the following formula to determine the sample size: N = PQ/ (D/T) ^2^, where P is the incidence rate of hypothermia; Q = 1-P; D is the tolerance, which is normally about 10% of P value; T is the statistic for the significance test. Considering imbalance among different regions nationwide, cluster sampling methodology, screening failure, drop-off, lost to follow up within 30 days and amount of funding to support this study, the final sample size was estimated to be 3000 subjects.

Randomized sampling was used to select the study subjects in each site. On one day prior to the surgery, investigator at each site screened subjects through a daily elective surgery information list (daily OR list for the next day) based on inclusion and exclusion criteria, and then generated a sub-list of all eligible patients. This sub-list then was submitted to our contract statisticians and 2 patients among the sub-list were randomly selected. Limited by work load, no more than 2 patients would be enrolled in each site on one day. Then the site investigator enrolled these eligible patients after they agreed and signed informed consent form.

### General anesthesia

All patients received either general anesthesia only or general anesthesia combined with regional anesthesia, depending on the preference of each hospital. The regimens of general anesthesia used were mostly propofol (2–2.5 mg/kg), fentanyl (2–4 μg/kg), and rocuronium (0.8–1 mg/kg) as induction, and sevoflurane (1.5–2 vol %) mixed with O_2_/N_2_O (50%/50%) for maintenance. Ropivacaine or lidocaine was used for regional anesthesia. The drugs such as non-steroidal anti-inflammatory drugs or others which may affect body temperature were not used in perioperative period, otherwise the cases were excluded from the study.

### Core temperature measurement

The primary outcome was inadvertent intraoperative hypothermia, defined as core temperature <36°C at any time during the perioperative period. The core temperature was assumed to be that of tympanic membrane temperature, since tympanic membrane temperature is easily obtained and has been validated to reflect core temperature. We applied an infrared tympanic membrane thermometer (Thermo Scan PRO-4000, Braun GmbH, Kronberg, Germany) to monitor temperature every 15 minutes before, during, and after operation. To reduce bias, the thermometer was calibrated and validated according to the manufacturer’s manual before use.

### Anesthesia and other risk factors

Patients’ demographic data were collected: age, gender, body mass index (BMI), medical history, and American Society of Anesthesiologists (ASA) physical status. BMI ≥25 kg/m^2^ was considered overweight or obese. The following data, considered potential risk factors associated with hypothermia, were also collected: baseline core temperature (prior to anesthesia), ambient temperature of the operating room, type of patient warming system, amount of intravenous fluid replacement, duration of anesthesia, and magnitude of surgery. Kinds of patient warming were categorized as passive (surgical draping, sheets, cotton blanket, etc.) or active (electric heating blanket, space heater, etc.). The magnitude of operations was classified according the classification reported previously [22].

### Postoperative follow up

Patients were followed up for 30 days after surgery. Investigators who conducted follow-up were not blinded to intraoperative anesthesia care. Length of stay in PACU, incidence/intensity of postoperative shivering, length of stay (LOS) in ICU, postoperative LOS in hospital, surgical site infection, and postoperative mortality rate within 30 days were recorded on the standard Case Report Form by one of the investigators.

### Statistical analysis

Descriptive analysis, including mean, standard deviation, frequencies, and percentages were presented, respectively. Student t test and chi-square were done for continuous variables and categorical variables, respectively. Multivariate logistic regression was applied to evaluate the potential risk factors for inadvertent intraoperative hypothermia. The potential confounders included in our regression model were age, gender, body mass index (BMI), ASA, BMI, baseline core temperature (prior to anesthesia), ambient temperature of the operating room, type of patient warming system used, amount of intravenous fluid replacement, duration of anesthesia, and magnitude of surgery. The results were presented as odds ratios together with a 95% confidence interval (95% CI). All analyses were performed with SAS 9.0 (SAS Institute, Cary, NC).

## Results

### Baseline characteristics of study population

A total of eligible 3132 patients from 28 hospitals nationwide were available in our analysis. The mean of age of these patients was 53.51 ± 13.80 years with BMI being 23.60±3.60 kg/m^2^. The majority of subjects was either ASA I (10.70%) or ASA II (77.62%) class. General surgeries (28.34%) and gynecologic surgeries (17.12%) were the two most frequent operation categories. Baseline data were summarized in Table 1.

**Table 1:** Patient demographics and anesthesia/surgery data (N = 3132).

Variables	Value
**total subjects**	3132
**Age**	
**mean± std (yr)**	53.51±13.80
**< = 65 n (%)**	2502 (79.89)
**< = 65 n (%)**	630 (20.11)
**Gender, n (%)**	
Male	1468 (46.87)
Female	1664 (53.13)
**BMI, n, mean± std**	23.60±3.60
**<25 (%)**	2147 (68.55)
**> = 25(%)**	985 (31.45)
**ASA, n (%)**	
1	335 (10.70)
2	2431 (77.62)
3	358 (11.43)
4	8 (0.25)
**Type of Surgery, n (%)**	
General Surgery	888 (28.35)
Hepatobillary Surgery	415 (13.25)
Peripheral Vascular Surgery	9 (0.29)
Cardiovascular Surgery	458 (14.62)
Thoracic Surgery	7 (0.22)
Orthopedics Surgery	332 (10.60)
Neurosurgery	1 (0.03)
Urology	431 (13.76)
Plastic Surgery	3 (0.10)
OB/GYN	533 (17.02)
Others	55 (1.75)
**Magnitude of Surgery**^1^**, n (%)**	
minor	12 (0.38)
intermediate	243 (7.76)
major	1235 (39.43)
major plus	1642 (52.43)
**Invasiveness of surgery**^2^**, n (%)**	
endoscopic surgery	1597 (50.99)
open surgery	1535 (49.01)
**Mode of Anesthesia, n (%)**	
general	2988 (95.40)
general + regional	144 (4.60)
**Total Anesthesia Time**^3^**, n, mean± std (h)**	
mean±std	2.97±1.49
min-max	1–14.78
median	2.67

^1^Magnitude of surgery as below: minor surgery: excision of lesion of skin; drainage of breast abscess, etc. Intermediate surgery: primary repair of inguinal hernia; excision of varicose vein(s) of leg; tonsillectomy/adenotonsillectomy; knee arthroscopy, etc. major surgery: total abdominal hysterectomy; endoscopic resection of prostate; lumbar discectomy; thyroidectomy, etc. major surgery plus: total joint replacement; lung operations; colonic resection; radical neck dissection; neurosurgery; cardiac surgery)

^2^ Arthroscopic, laparoscopic, etc.

^3^ Time from induction to discontinuation of anesthetic agents

### Incidence of hypothermia and patient outcomes

**Fig 1:**
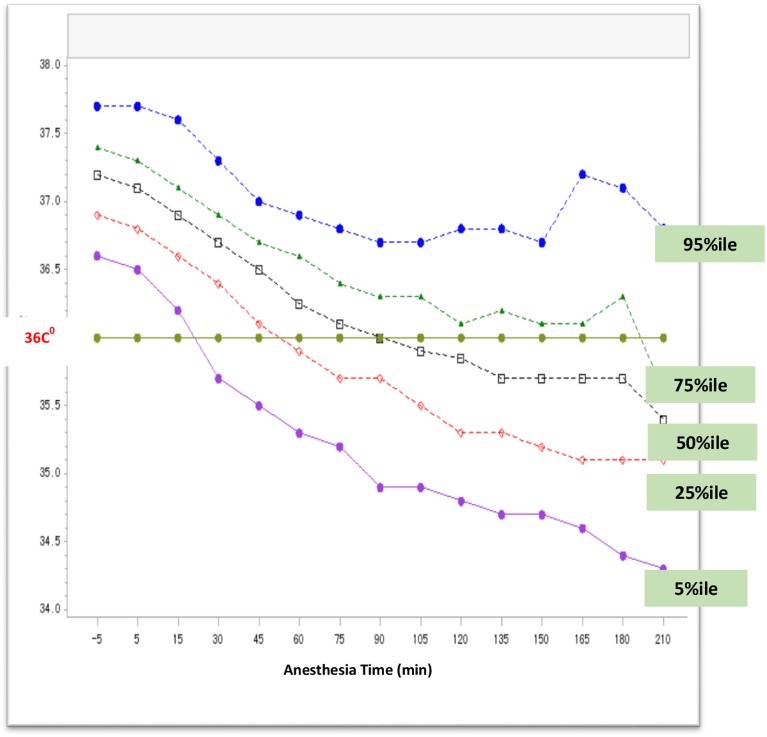
Change of intraoperative core temperature during operations. Patients’ core temperature was measured at the tympanic membrane beginning every 15 minutes after the induction of anesthesia until the end of the operation. A total of 3132 subjects were enrolled in the study; each curve represents a connection of 5 percentiles, 25 percentile, 50 percentile, 75 percentile and 95 percentile of core temperature at a specific time point. The time interval is 15 minutes.

The overall incidence of inadvertent intraoperative hypothermia was 44.3% (Fig 1). The cumulative incidence rates of hypothermia within 1hr, 2hr, 3hr and 4hr following induction of anesthesia were 17.8%, 36.2%, 42.5% and 44.1% respectively. All patients were passively warmed by covering of surgical draping, sheets or cotton blankets, whereas only 14.2% of patients received additional active warming using space heaters or electric heater or electronic blankets (Table 2).

**Table 2:** Incidence of intraoperative hypothermia, patient warming and clinical outcome (N = 3132).

Variables	Value
**Overall Incidence of Hypothermia (T< 36.0°C)**	1386 (44.25)
**Cumulative Incidence of hypothermia**	
within 1 hour	17.8%
within 2 hours	36.2%
within 3 hours	42.5%
within 4 hours	44.1%
**Baseline core temperature prior to anesthesia (°C)**	
mean±std	37.2±0.3
min-max	36.0–38.0
median	37.2
**OR ambient temperature (°C)**	
mean±std	22.9±1.4
min-max	17.0–29.6
median	23
**Patient receiving intraoperative passive warming^1^, n (%)**	3132(100.0)
**Patient receiving intraoperative active warming^2^, n (%)**	443 (14.17)
**Blood transfusion**	
autologous	
n (%)	75 (2.4)
mean±std (ml)	519.8±372.2
median (ml)	447
min-max (ml)	0–2250
allogeneic	
n (%)	317 (10.1)
mean±std (ml)	735.2±500.6
median (ml)	600
min-max (ml)	50–3540
**Perioperative IV fluid**	
n (%)	3132 (100)
mean±std (ml)	1768.3±819.3
median (ml)	1600
min-max (ml)	250–10200
patients receiving prewarmed IV fluid, n (%)	649 (20.7)
patients receiving unwarmed IV fluid, n (%)	2483 (79.3)
**Intraoperative irrigation fluid**	
n (%)	2108 (67.3)
mean±std (ml)	1879.9±8194.5
median (ml)	1000
min-max (ml)	20–33000

^1^ passive warming includes comforter, blanket etc.

^2^active warming includes electric heated blanket, space heater etc.

Among 3132 participating patients, 1386 (44.3%) experienced hypothermia and 1746 (55.7%) were normothermic. Compared to normothermic patients, hypothermic patients had a greater prevalence (17.53% vs. 5.04%, P<0.0001) and more intense (level 3 shivering: 5.63% vs. 1.55%, P<0.0001; level 4 shivering: 1.73% vs. 0.23%, P<0.0001) shivering. The length of PACU stay was significantly longer in hypothermic patients than those in normothermic patients (1.77±3.07 h vs. 1.25 ± 1.47h, P<0.0001). Moreover, hypothermic patients had longer hospital stay postoperatively than normothermic patients (postoperative bed days: 16.97 ± 8.93 days vs. 14.99 ± 8.25 days, P<0.0001). Additionally, hypothermic patients had more chance to be admitted to ICU than normothermic ones (postoperative ICU admit rate: 10.03% vs. 4.64%, P<0.0001). However, our data indicated there was no significant difference between hypothermia and normothermia in surgical site infection (2.41% vs. 2.59%), or 30-day postoperative mortality (0.35% vs. 0.44%) (Table 3).

**Table 3:** Outcome of hypothermia and normothermia.

	Total (n = 3132)	Hypothermia (n = 1386, 44.3%)	Normothermia (n = 1746, 55.7%)	P value
**Postoperative Shivering, n (%)**				
without shivering	2801(89.43)	1143(82.47)	1658(94.96)	<0.0001
with shivering	331 (10.57)	243 (17.53)	88 (5.04)	
Intensity of Postoperative Shivering ^a^, n (%)				
1	83(2.65)	56(4.04)	27(1.55)	
2	109(3.48)	85(6.13)	24(1.37)	
3	111(3.54)	78(5.63)	33(1.89)	
4	28(0.89)	24(1.73)	4(0.23)	
**Length of PACU stay (hour)**				
mean± std	1.47(2.33)	1.77(3.07)	1.25(1.47)	<0.0001
min- max	0–22.50	0.07–22.50	0–20.17	
median	1.08	1.17	1.07	
**Length of postoperative hospital stay (day)**				
mean± std	15.86±8.61	16.97±8.93	14.99±8.25	<0.0001
min- max	2.00–99.00	3.00–86.00	2.00–99.00	
median	14	15	14	
**Postoperative ICU admit, n (%)**	220(7.02)	139(10.03)	81(4.64)	<0.0001
**Length of ICU stay (day)**				
mean± std	2.31±3.26	2.46±3.36	2.0±3.07	0.3615
min- max	0–29	0–29	1–26	
median	1	1	1	
**Surgical Site Infection (SSI)**				
number of patients	3032	1372	1738	0.7446
missing subjects	22	14	8	
SSI, number (%)	78(2.51)	33 (2.41)	45 (2.59)	
**Mortality within postoperative 30 days**				
number of patients	3100	1379	1727	0.486
missing subjects	21	13	8	
death, n (%)	11(0.35)	6 (0.44)	5 (0.29)	

^a^ intensity of shivering shown as below

0 = no shivering

1 = no visible muscle activity but piloerection, peripheral vasoconstriction, or both are present

2 = muscular activity in only one muscle group

3 = moderate muscular activity in more than one muscle group but no generalized shaking

4 = violent muscular activity that involves the whole body.

### Risk factors significantly contributed to hypothermia

Based our previous studies and literature review, we assessed risk factors significantly contributed to hypothermia by multiple logistic regression. As results of this study (Table 4), the following risk factors associated with hypothermia are either significantly protective or predisposing. Protective factors are active warming (OR = 0.46, 95% CI 0.26–0.81), BMI ≥ 25 (OR = 0.54, 95% CI 0.45–0.65), higher baseline core temperature (OR = 0.04, 95% CI 0.03–0.06) and higher ambient temperature (OR = 0.83, 95% CI 0.78–0.88). Meanwhile, predisposing factors include major-plus surgery (OR = 1.49, 95% CI 1.23–1.79), longer duration of anesthesia (>2 h) (OR = 2.60, 95% CI 2.09–3.24), non-endoscopic surgery (OR = 1.30, 95% CI 1.10–1.54), IV fluid > 1000ml (OR = 1.57, 95%CI 1.22–1.74), and intraoperative irrigation fluid > 500ml (OR = 1.47, 95%CI 1.24–1.74).

**Table 4:** Risk factors associated with intraoperative hypothermia.

	Crude OR(95%CI)	P value	Adjusted OR^1^ (95%CI)	P value
**Age**				
< = 65	reference		reference	
>65	1.471(1.234–1.752)	<0.0001	1.14(0.92–1.40)	0.2330
**Gender**				
Male	reference		reference	
Female	0.638(0.553–0.7435)	<0.0001	1.10(0.93–1.31)	0.278
**ASA**				
1 and 2	reference		reference	
≥3	1.119(0.900–1.393)	0.3118	0.87(0.67–1.13)	0.304
**Magnitude of Surgery**				
Minor/Intermediate Surgery	0.746(0.557–0.999)	<0.0001	0.89(0.63–1.24)	0.0562
Major Surgery	reference		reference	
Major Plus Surgery	1.941(1.669–2.258)	<0.0001	1.49(1.23–1.79)	<0.0001
**Anesthesia**				
General Anesthesia alone	reference		reference	
Anesthesia (combined)	1.237(0.886–1.729)	0.2121	0.99(0.67–1.46)	0.943
**IV fluid replacement (>1000ml)**				
≤1000ml	reference		reference	
>1000ml	2.306(1.896–2.805)	<0.0001	1.57(1.22–1.74)	0.0004
**Intraoperative irrigation**				
≤500ml	reference		reference	
>500ml	1.592(1.381–1.835)	<0.0001	1.47(1.24–1.74)	<0.0001
**Duration of Anesthesia**				
≤2h	reference			reference
>2 h	2.64(2.24–3.11)	<0.0001	2.60(2.09–3.24)	<0.0001
**Endoscopic Surgery**				
yes	reference		reference	
no	1.09(0.95–1.26)	0.0595	1.30(1.10–1.54)	0.0026
**Patient Warming**				
passive warming	reference		reference	
active warming	1.11(0.91–1.35)	0.3198	0.62(0.49–0.80)	<0.0001
**Baseline core temperature before anesthesia (C°)**	0.06(0.05–0.08)	<0.0001	0.04(0.03–0.06)	<0.0001
**BMI**				
> = 25	reference		reference	
<25	0.57(0.48–0.66)	<0.0001	0.54(0.45–0.65)	<0.0001
**OR ambient temperature (C°)**	0.84(0.80–0.88)	<0.0001	0.83(0.78–0.88)	<0.0001

OR, Odds Ratio

Significant level indicates as P<0.05

^1^ adjusted OR were presented after adjusting all the variables above

## Discussion

Perioperative hypothermia has been considered a common but preventable adverse event so that its avoidance has been recommended and incorporated into various clinical guidelines [23]. For example, the Surgical Care Improvement Project suggested a final intraoperative temperature above 36°C and/or use of active over-body warming whether or not core temperature reached 36°C [24]. In the United States, practice guidelines recommend that all surgical patients be actively warmed. [20,25] Active warming is also suggested but relatively uncommon in many western developed countries [20,21]. However, developing countries like China, few patients are actively warmed. In 2013–2014, we conducted a regional survey among 24 hospitals in Beijing [22]. The 24 participating hospitals were randomly selected from a total of 74 hospitals in the City of Beijing, including teaching hospitals and community hospitals. However, due to technical feasibility and limited research funding, the national survey did not randomly select participating hospitals. Instead, we only enrolled 28 representative major academic teaching medical centers to cover the most geographical areas in China. In the national study, our results indicate that active warming accounted for only 14.2% of total subjects and the overall rate of hypothermia is as high as 44.3%. Hypothermic patients were associated with higher ICU admission rates, longer PACU stay, prolonged recovery time, and longer postoperative hospital stay. The national hypothermia incidence was slightly higher than Beijing’s rate (39.9%) in our previous study, indicating that hypothermia is truly common in China. Moreover, similar to the results of Beijing Regional Survey, national data also showed intraoperative active warming, higher body mass index (BMI), higher baseline core temperature before anesthesia, and higher ambient temperature help maintain intraoperative normothermia. In contrast, major operations, longer duration of anesthesia (>2h), intravenous unwarmed fluid infusion >1000ml and open surgery significantly increase the risk of hypothermia [26]. Interestingly, we found incident rates varied by geographic sites: many participating sites in the north had below average rates, while most sites in the south showed above average incident rates. The geographic variance of incidence rate may be related to differences in baseline OR temperature. Perhaps due to hot weather, OR ambient temperature was usually set lower in the south than in the north.

Perioperative hypothermia has been reported to be associated with the risk of infection complications ([12–15,27]. However, our national data did not show significant results of such within postoperative 30 days. There could be several reasons: First, this study could be still underpowered to look to significant difference in surgical site infection (SSI) between hypothermia and normothermia; Secondly, general surgery and obstetric/gynecologic surgeries (OB/GYN) had the first and second largest numbers of patients, in which half of patients (50.99%) underwent minimal invasive procedure (endoscopic surgery) with small incisions; Third, preoperative and postoperative antibiotics are routinely used in China, even in clean surgery; Fourth, due to current questionable quality evaluation standard, performance review and reimbursement policy in China, SSIs are more likely underreported.

Like most observational studies, there are a few limitations in our study. First, unlike Beijing Regional Study, multistage randomized sampling was not technically feasible in this study. As a matter of fact, we chose hospitals nationwide largely based on the convenience of the principle investigator (PI) and research capacity of his / her team rather than randomization. The participating hospitals are all major academic teaching medical centers with good capacity of conducting such a research, which also limited the generation of our results. We did try to cover as many of the geographic sites shown in our PI name list. Therefore, the sample patient population evaluated in this study does not exactly reflect a nationally representative patient population. Randomized sampling was applied in each site through daily OR list so those patients are well representative of that particular hospital. Secondly, awareness of hypothermia and knowledge of its adverse effects could, to some extent, make some of investigators/sites take extra action against temperature drop, i.e. addition of heating blanket or space heater or warmed fluid in their practice to keep normothermia. Therefore, the actual incident rate of hypothermia could be underestimated.

Temperature is one of the vital signs following blood pressure, heart rate and respiration rate. Many clinical practice guideline and recommendation in developed countries have clearly specified the goal and operation protocol of temperature management. However, in 1990s or earlier, a shortage of health care resources and insufficient social economic development in China hindered the action of temperature management. Today, under the context of rapid development of social economics in China, scarcity of health care resource is no longer the main cause or excuse for ignorance of temperature management. During our study, based on our informal interview and communication with participating investigators, unawareness of adverse consequence related to hypothermia and neglect of temperature may be the primary cause of hypothermia. On the other hand, either charge code or reimbursement policy on perioperative actively patient warming are not available in China, which also lead to extremely low utilization of patient warming system.

In summary, we concluded that the incidence of intraoperative hypothermia in China is high, and the rate of active warming of patients during operation is low. Hypothermia is associated with more postoperative shivering, more ICU admit, and longer postoperative hospital days.
